# Potential effect of dietary zinc intake on telomere length: A cross-sectional study of US adults

**DOI:** 10.3389/fnut.2022.993425

**Published:** 2022-11-16

**Authors:** Huanchen Shi, Xiaoxuan Li, Haihong Yu, Wanting Shi, Yue Lin, Yunping Zhou

**Affiliations:** ^1^School of Medicine, Qingdao University, Qingdao, China; ^2^School of Nursing, Qingdao University, Qingdao, China

**Keywords:** dietary zinc intake, telomere length, oxidative stress, inflammation, aging, NHANES

## Abstract

**Background:**

Telomere length, which is related to chronic diseases and premature mortality, is influenced by dietary factors. Zinc is known as a dietary antioxidant micronutrient, however, its impact on telomere length remains unclear.

**Objective:**

We aimed to examine the potential effect of dietary zinc intake on telomere length among middle-aged and older individuals in the US.

**Materials and methods:**

Our study included 3,793 US participants aged 45 years and older from the 1999 to 2002 National Health and Nutrition Examination Survey (NHANES). 24-h dietary recall interviews were employed to evaluate zinc consumption. Leukocyte telomere length was assessed by real-time quantitative polymerase chain reaction (qPCR). We adopted generalized linear models to investigate the effect of dietary zinc intake on telomere length, and subgroup analyses were further applied. We further evaluated the dose-response relationship using restricted cubic spline analysis.

**Results:**

Among the 3,793 participants, the average telomere length was 0.926 ± 0.205 (T/S ratio) or 5509.5 ± 494.9 (bp). After adjusting for major confounders, every 5 mg increment in dietary zinc consumption was related to 0.64% (95% CI: 0.17%, 1.10%) longer telomere length. In the subgroup analyses, significant relationships were found in females (Percentage change: 1.11%; 95% CI: 0.48%, 1.75%), obese (Percentage change: 0.88%; 95% CI: 0.26%, 1.50%), and low energy intake individuals (Percentage change: 0.99%; 95% CI: 0.51%, 1.46%). Additionally, we revealed a positive linear relationship between dietary zinc intake and telomere length (*P* for non-linearity = 0.636).

**Conclusion:**

Our study revealed that elevated dietary zinc intake was significantly related to longer telomere length among adults aged 45 years and older in the US. And the association was more pronounced in females, obese, and low energy intake individuals.

## Introduction

Zinc, an essential dietary micronutrient necessary for normal growth and development (1), performs a wide variety of physiological and cellular functions through its association with metallothioneins (MTs). The MTs are cysteine-rich proteins that bind to metal ions such as zinc and mainly function as antioxidants or radical scavengers (2). Studies have identified antioxidant, anti-inflammatory, anti-tumor, and immune-modulatory properties of zinc (3–5). Red meat, poultry, fish, and dairy products are primary dietary sources of zinc (6). However, approximately 17.3% of people worldwide do not consume adequate zinc (7). And patients with malabsorption syndrome, liver disease, chronic renal disease, sickle cell disease, and other chronic illnesses have higher risks of zinc deficiency (8). Zinc deficiency is related to a higher risk of various disease conditions, such as cardiovascular diseases, type 2 diabetes mellitus, growth retardation, cognitive impairment, infectious diseases, male hypogonadism, skin changes, delayed wound healing, and so on (8, 9). In contrast, adequate zinc consumption could benefit people with chronic metabolic disease, diabetes, and cardiovascular diseases (1, 10). Hence, adequate dietary zinc intake is required to maintain human health.

Telomeres are highly conserved structures at the distal ends of eukaryotic chromosomes comprising repetitive DNA sequences (TTAGGG) (11). Generally, they are shortened in each replication of somatic cells (12), while in embryonic and adult stem cells, germ cells can be elongated by a telomerase complex composed of telomerase RNA (TR) and telomerase reverse transcriptase (TERT) (11). Telomeres protect chromosome ends from deteriorating and fusing, which is important for maintaining genomic stability and chromosomal structural integrity (13). It has been reported that oxidative stress and inflammation could lead to telomere shortening (14, 15), thereby increasing the incidences of aging-related disorders (16, 17). To pinpoint the critical causes of telomere shortening is propitious for ascertaining the pathophysiological mechanisms behind these chronic diseases. Previous research indicated that the intake of antioxidant and anti-inflammatory food could counteract this shortening and contribute to longer telomere length (18, 19). As an important dietary trace element, zinc is known for its powerful antioxidant and anti-inflammatory properties. Therefore, it’s reasonable to hypothesize that elevated zinc intake can help preserve telomere length, which is beneficial to a longer life expectancy. Although some studies have investigated the associations between dietary elements and telomere length, epidemiological studies elucidating the associations of zinc consumption with telomere length are scarce.

To fill this knowledge gap, the present study was designed to explore the potential effect of zinc intake on telomere length among middle-aged and older individuals in a large population-based dataset, the National Health and Nutrition Examination Survey (NHANES).

## Materials and methods

### Study populations

The National Health and Nutrition Examination Survey, a cross-sectional survey aiming to evaluate the nutritional status and health of the US population, is administered by the US government. NHANES applies a complex, multistage, multifaceted sample strategy to acquire a nationally representative sample. Data about the participants’ demographics, education, socioeconomic status, dietary information, lifestyle, and medical conditions are gathered as part of the NHANES survey. Additionally, comprehensive medical examinations are carried out and specimens are collected from study participants for laboratory examinations. NHANES data are released in 2-year cycles. This study was approved by the National Center for Health Statistics Research Ethics Review Board and written informed consent was provided by each participant. The ethical approval code for NHANES data collection of 1999–2002 is #98-12.

**FIGURE 1:**
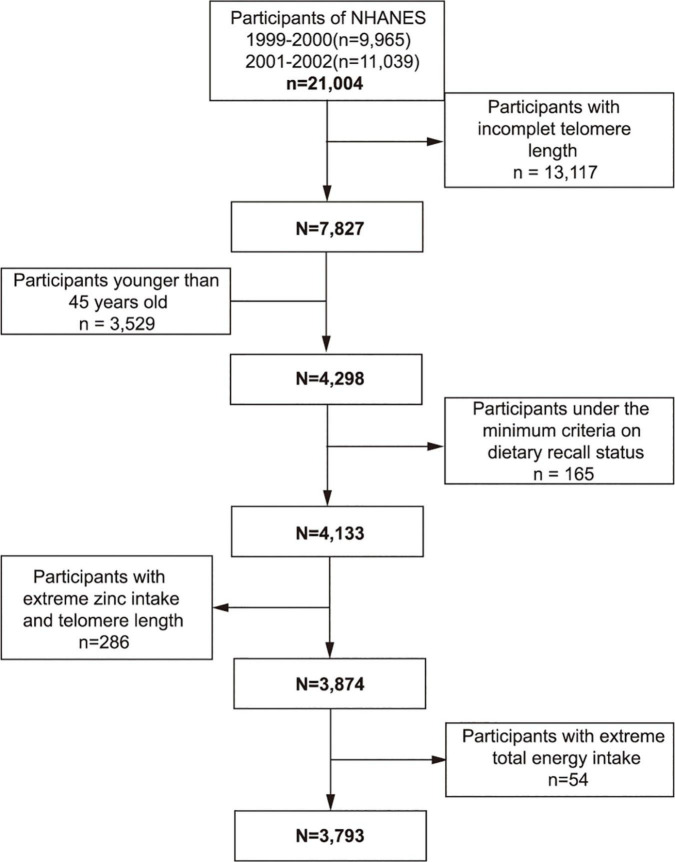
Study flowchart. Flowchart showing study participant selection. Of 21,004 participants in the 1999–2002 National Health and Nutrition Examination Survey (NHANES), 3,793 remained after fulfilling inclusion and exclusion criteria.

From the 1999–2002 NHANES, 7,827 individuals who had available data on telomere length and dietary zinc intake constituted the study sample. Participants below the age of 45 (*n* = 3,529) were excluded from the study. People were not included in the study if they were under the minimum criteria of dietary recall status (*n* = 165) or if their telomere length and dietary zinc intake exceeds 1.5 interquartile ranges below the 25th percentile or above the 75th percentile (*n* = 286). We also excluded individuals whose total energy intake was less than 500 or above 5,000 kcal/per day for females and less than 500 or above 8,000 kcal/per day for males (*n* = 54). Finally, this cross-sectional study encompassed 3,793 participants in total (Figure 1).

### Telomere length measurement

National Health and Nutrition Examination Survey obtained blood samples from each participant. The telomere length quantification was conducted in the laboratory of Dr. Elizabeth Blackburn at the University of California, San Francisco. Real-time quantitative polymerase chain reaction (qPCR) was used to quantify telomere length relative to standard reference DNA (T/S ratio) in the blood leukocytes. The quantity of telomere repeats (T) to the relative quantity of single copy gene (S) (T/S ratio) should be proportional to the average telomere length in all experimental DNAs. β-globin/36B4 was the single copy gene used as a control to standardize input DNA. More details about the telomere length measurement were described previously (20, 21). Each sample was assigned to duplicate wells in 96-well plate and was assayed three times on three different days. To normalize between-run variability, each assay plate contained eight control DNA. After excluding possible outliers of the samples, the mean and standard deviation of the T/S ratio were then calculated normally. The inter-assay coefficient of variation was 6.5%. To convert T/S ratio to base pairs (bp), the formula was applied: 3,274 + 2,413 × (T/S).

### Zinc intake assessment

Dietary zinc intake was assessed *via* 24-h recall administered by a trained interview from what we eat in America survey which was performed in the Mobile Examination Center (MEC). The NHANES computer-assisted dietary interview (CADI) system recorded the types and amounts of food and drink consumed by the subjects during the 24 h preceding the interview. According to the study protocol, all participants were randomly assigned to data collecting exam sessions in the morning or the afternoon/evening. The dietary consumption of zinc and other nutrients was calculated based on the University of Texas Food Intake Analysis System and US Department of Agriculture Survey Nutrients Database. Nutrients acquired from dietary supplements or medications were excluded from nutrient estimates. More details about data gathering were available on the NHANES website.^1^

### Covariates assessment

The following covariates were included in our research: age, sex, race, education background, cigarette smoking, alcohol drinking, hypertension, household income, diabetes, BMI, and overall energy intake. A questionnaire or physiological and biochemical test was used to determine the participants’ basic characteristics and health status. Our analysis classified the race into non-Hispanic whites, non-Hispanic blacks, Mexican Americans, and others. Education level was categorized as below high school, high school, and above high school. Smokers were defined as someone who had smoked at least 100 cigarettes in their lifetime. The definition of a drinker was an individual who had consumed alcohol at least 12 times a year. The use of antihypertensive agents, or the mean systolic blood pressure higher than 130 mmHg, or the mean diastolic blood pressure above 80 mmHg were all defined as hypertension. Diabetes was identified according to self-reported diabetes history.

### Statistical analysis

The Kolmogorov–Smirnov normality tests were performed to test the normality of continuous variables. The continuous variables were described by means ± standard deviations, while the categorical variables were described by counts (percentages). The length of the telomere is transformed logarithmically to approximate the normal distribution. Generalized linear model was employed to measure the relationship between dietary zinc intake and telomere length. In our research, four models were employed, with major confounders being adjusted based on earlier studies. Model 1 is the unadjusted model without controlling for confounding variables. Age, sex, race, education, and family income were adjusted in model 2. In model 3, we further included smoking, alcohol drinking, BMI, diabetes, and hypertension. Model 4 was further adjusted for overall energy intake. We calculated the correlation between dietary zinc intake and other trace elements and vitamins, and the results were shown in Supplementary Table 1. We found a high correlation between dietary zinc intake and dietary iron, copper, magnesium, selenium, riboflavin intake, respectively (correlation ≥ 0.50, *p*-value < 0.05). To avoid the negative effect of multicollinearity on the regression model, we did not adjust the variables mentioned above. To interpret the regression models better, the percentage change in telomere length for every 5 mg incremental increase of the dietary zinc consumption was calculated using the formula [e^(5 × β)^ − 1] × 100%, with 95% confidence intervals (CI) estimated as (e^[5 × (β ± 1.96 × SE)]^ − 1) × 100%, where β and SE represent the estimated regression coefficient and the standard error, respectively.

Since prior studies have revealed that sex, BMI, and energy intake were related to telomere length (22, 23), we conducted subgroup analyses to explore the potential modifiers of BMI, overall energy intake and the participants’ sex on telomere length. The participants in our study were dichotomized into three subgroups depending on sex (male; female), the cutoff value of obesity (non-obesity: <30 kg/m^2^; obesity: ≥30 kg/m^2^), median overall energy intake (low energy intake: <1,726 kcal; high energy intake: ≥1,726 kcal). The relationship of dietary zinc consumption with telomere length was analyzed in each subgroup. We also investigated the interaction effects using the Wald test. Additionally, restricted cubic splines were applied to estimate the dose-response relationship between dietary zinc intake and telomere length across all participants, and we placed three knots at the 5th, 50th, and 95th percentile, respectively. In our study, the data were weighted to provide nationally representative estimates. Stata 14.0 (Stata Corporation, College Station, TX, USA) was used to carry out each statistical analysis. The statistical significance of the study was defined as a two-sided *p*-value less than 0.05.

## Results

Table 1 displayed the basic characteristics of the participants. The sample was comprised of 3,793 participants, out of which 1,909 (46.9%) were males and 1,884 (53.1%) were females, 1,229 (33.7%) were obese and 2,419 (66.3%) were non-obese. 2,042 (54.7%) of the participants were smokers and 2,383 (67.4%) were drinkers. The prevalence of hypertension and diabetes in the present study population was 58.6% and 89.0%, respectively. The average age of the sample was 60.2 ± 11.3 years, mean dietary zinc intake was 9.6 ± 4.7 mg/day, and mean telomere length was 0.926 ± 0.205 (T/S ratio) or 5509.5 ± 494.9 (bp). The median overall energy intake was 1,726 kcal.

**TABLE 1:** Characteristics of the participants (*N* = 3,793).

Characters	Mean ± SD/*N* (%)
Age, years	60.2 ± 11.3
**Sex, %**	
Male	1,909 (46.9)
Female	1,884 (53.1)
**Race, %**	
Non-Hispanic white	2,125 (79.1)
Non-Hispanic black	588 (7.5)
Mexican American	806 (3.9)
Other Hispanic	184 (6.0)
Other race	90 (3.5)
**Education, %**	
Below high school	1,441 (23.9)
High school	844 (25.7)
Above high school	1,504 (50.4)
**Cigarette smoking, %**	
No	1,745 (45.3)
Yes	2,042 (54.7)
**Alcohol drinking, %**	
<12 times/year	1,302 (32.6)
≥12 times/year	2,383 (67.4)
**Hypertension, %**	
No	1,379 (41.4)
Yes	2,340 (58.6)
**Household income, %**	
<$20,000	982 (21.5)
≥$20,000	2,364 (78.5)
**Diabetes, %**	
No	556 (11.0)
Yes	3,160 (89.0)
**BMI, %**	
<30 kg/m^2^	2,419 (66.3)
≥30 kg/m^2^	1,229 (33.7)
**Overall energy intake, %**	
<1,726 kcal	1,896 (44.8)
≥1,726 kcal	1,897 (55.2)
Zinc intake, mg	9.6 ± 4.7
Telomere length, T/S ratio	0.926 ± 0.205
Telomere length, bp	5509.5 ± 494.9

SD, standard deviation; BMI, body mass index.

Table 2 presented the correlation between dietary zinc intake and telomere length in all participants and subgroups. For all respondents, every 5 mg increment of zinc consumption was linked with 0.69% (95% CI: 0.31%, 1.07%) longer telomere length in the unadjusted model. After adjusting for differences in age, sex, race, educational levels, and household income, every 5 mg increment of zinc consumption was related to 0.62% longer telomere length (95% CI: 0.18%, 1.06%). After further adjusting for cigarette smoking, alcohol drinking, BMI, diabetes, and hypertension, the result still indicated a remarkable positive correlation (Percentage change: 0.64%; 95% CI: 0.17%, 1.10%). However, after additional adjustment for overall energy intake, the result was insignificant.

**TABLE 2:** The association between dietary zinc intake and telomere length among all participants and subgroups.

Participants	*N*	Telomere length (bp)	Models	Percentage change (%) and 95% CI	*P*-value	^a^P for interaction
All participants	3,793	5509.5 ± 494.9	Model 1	0.69 (0.31, 1.07)	0.001	
			Model 2	0.62 (0.18, 1.06)	0.010	
			Model 3	0.64 (0.17, 1.10)	0.012	
			Model 4	0.52 (−0.01, 1.06)	0.065	
Sex subgroup						0.054
Female	1,884	5545.4 ± 493.6	Model 1	1.11 (0.55, 1.68)	0.001	
			Model 2	1.10 (0.52, 1.68)	0.001	
			Model 3	1.11 (0.48, 1.75)	0.002	
			Model 4	0.82 (0.06, 1.58)	0.042	
Male	1,909	5474.1 ± 493.8	Model 1	0.56 (0.05, 1.08)	0.041	
			Model 2	0.19 (−0.32, 0.69)	0.472	
			Model 3	0.20 (−0.31, 0.72)	0.440	
			Model 4	0.24 (−0.32, 0.81)	0.402	
BMI subgroup						0.680
Obese (≥30 kg/m^2^)	1,229	5530.7 ± 488.6	Model 1	0.67 (0.15, 1.20)	0.017	
			Model 2	0.92 (0.34, 1.51)	0.004	
			Model 3	0.88 (0.26, 1.50)	0.009	
			Model 4	0.75 (−0.12, 1.63)	0.100	
Non-obese (<30 kg/m^2^)	2,419	5502.7 ± 493.5	Model 1	0.73 (0.25, 1.22)	0.006	
			Model 2	0.52 (−0.03, 1.08)	0.074	
			Model 3	0.51 (−0.08, 1.09)	0.099	
			Model 4	0.40 (−0.18, 0.99)	0.186	
Energy subgroup						0.598
Low energy intake (<1,726 kcal)	1,896	5490.7 ± 494.9	Model 1	0.68 (0.23, 1.13)	0.006	
			Model 2	0.94 (0.46, 1.42)	0.001	
			Model 3	0.99 (0.51, 1.46)	0.000	
			Model 4	0.79 (0.19, 1.40)	0.015	
High energy intake (≥1,726 kcal)	1,897	5528.4 ± 494.4	Model 1	0.46 (−0.17, 1.08)	0.164	
			Model 2	0.42 (−0.26, 1.10)	0.234	
			Model 3	0.42 (−0.27, 1.10)	0.276	
			Model 4	0.37 (−0.35, 1.10)	0.326	

CI, confidence interval; BMI, body mass index; Model 1 was a crude model, including zinc intake; Model 2 was further adjusted for age, sex, race, educational levels, and household income; Model 3 was additionally adjusted for cigarette smoking, alcohol drinking, BMI, diabetes, and hypertension; Model 4 was further adjusted for overall energy intake.

^a^*P*-value for the interaction of Model 4.

Furthermore, subgroup analyses were conducted to evaluate whether such relationships were affected by sex, BMI, or overall energy intake. After controlling for age, sex, race, educational levels, household income, smoking, alcohol drinking, BMI, diabetes, and hypertension, significant relationships were observed in females, obese and low energy intake participants. Every additional 5 mg of dietary zinc consumption was related to 1.11% (95% CI: 0.48%, 1.75%), 0.88% (95% CI: 0.26%, 1.50%), and 0.99% (95%CI: 0.51%, 1.46%) longer telomere length in females, obese, and low energy intake participants, respectively. After further controlling for overall energy intake, the association between dietary zinc consumption and telomere length was still significant in females (Percentage change: 0.82%; 95% CI: 0.06%, 1.58%) and low energy intake participants (Percentage change: 0.79%; 95% CI: 0.19%, 1.40%). Besides, the data showed no significant interaction effect between dietary zinc consumption and sex, BMI, or energy intake (*P* for interaction was 0.054, 0.680, and 0.598, respectively).

**FIGURE 2:**
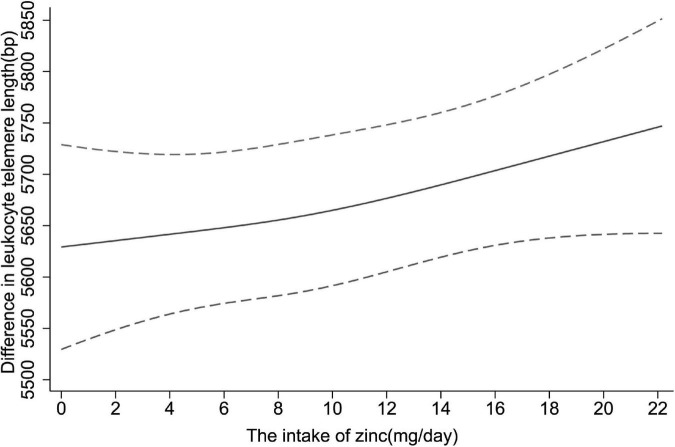
The dose-response relationship between dietary zinc intake and telomere length. Point estimates (solid line) and 95% confidence intervals (dashed lines) were estimated by restricted cubic splines analysis with knots placed at the 5th, 50th, and 95th percentile (minimum as the reference). Models were adjusted for age, sex, race, educational levels, household income, cigarette smoking, alcohol drinking, BMI, diabetes, hypertension and overall energy intake.

Moreover, restricted cubic spline was applied to explore the dose-response relationship between dietary zinc consumption and telomere length. In Figure 2, a significantly positive linear relationship was observed in the whole participants (*P* for non-linearity = 0.636).

## Discussion

In this cross-sectional survey, we evaluated the effect of dietary zinc intake on telomere length in a randomly selected NHANES sample of 3,793 adults aged 45 years and older. The findings revealed that in model 1, 2, and 3, dietary consumption of zinc was significantly and positively correlated with telomere length in all participants. In addition, this correlation was more significant in females, obese and low energy intake participants. Moreover, we further controlled for total energy intake in model 4 to exclude the effect of energy intake on telomere length. The results indicated that in model 4, associations between dietary zinc intake and telomere disappeared in all participants, but remained significant in females and low energy intake subgroup. Besides, there was no interaction effect between zinc consumption and overall energy intake, which suggested that low dietary zinc intake was not just a sign of low energy intake and the observed associations were independent of overall energy intake.

Telomere length is considered to be a credible biomarker of biological age and life span (24). Apart from chronological age, telomere length is also influenced by dietary factors. Previous studies indicated that intake of foods with antioxidant and anti-inflammatory properties such as some vitamins, fiber, magnesium, copper, and selenium contributed to longer telomere length (22, 25, 26). On the contrary, risk factors for telomere shortening included consumption of processed meat, caffeine, sugary drinks, and so on (27–29). Telomere shortening increased the risk of age-related chronic diseases and even cancers, which markedly curtailed life expectancy (16, 17, 30). Our findings were the first to exhibit a significant positive association between dietary zinc consumption and telomere length, which provided new insights into the mechanism of aging and chronic diseases.

Prior studies have demonstrated the role of zinc in the maintenance of genome stability and integrity (31–33), and our findings are consistent with the assumptions of the existing literature. Liu et al. found that certain concentration of zinc was conducive to preserving the telomere length of hepatocytes L-02 (33). And Sharif et al. showed that zinc at physiological concentrations helped to maintain the genomic stability of WIL2-NS cells, whereas the lower and higher concentrations of zinc would induce increased cellular death (32). However, very few experiments have directly revealed the effect of zinc on maintaining telomere length, and more experiments are needed.

Besides, previous studies have also reported the relationship between serum zinc and telomere length, but the association is still controversial. One study demonstrated that high serum zinc level might slow telomere attrition in male adults (34), but prior research also found an inverse association between serum zinc intake and telomere length in children (35, 36). Future studies are required to confirm the effect of serum zinc on telomere length. Moreover, it was reported that plasma zinc level was vulnerable to insufficient dietary intakes (37), while a moderate increase in dietary zinc intake would improve zinc absorption and the repair of DNA strand breaks, but would not change plasma zinc (38). Zinc deficiency and excess may both relate to adverse health outcomes. Hence, we speculate that moderately increased dietary zinc intake is propitious to optimize health effects. Further research is needed to determine the optimal range of dietary zinc intake.

Although the underlying mechanism of the relationship between dietary consumption of zinc and telomere length has not yet been elucidated, it has several possible explanations. Firstly, the DNA repeats that constitute telomeres include high contents of guanines, which are sensitive to oxidative stress (39). Oxidative stress could induce single strand breaks at telomeres directly or indirectly by interfering with DNA repair and thus shortening telomere length (40). Importantly, zinc has shown powerful antioxidant effects, and thus might preserve telomere length through its antioxidant effects. Secondly, increased production of pro-inflammatory cytokines was associated with telomere shortening (41). Zinc could inhibit transcription factor NF-κB by upregulating the zinc-finger protein ZA20 (42), resulting in suppressed secretion of pro-inflammatory enzymes, cytokines, and adhesion molecules (41, 43). Thirdly, DNA polymerases, RNA polymerases and reverse transcriptases are zinc-dependent enzymes (44, 45). A previous study reported that adding additional zinc in the cell-culture medium could increase activity of telomerase (46). Moreover, zinc is an essential part of Human Tankyrase1 (TANK1), which is a member of the poly ADP-ribose polymerases involved in DNA repair at DNA damage sites (47). Dietary zinc deficiency was shown to increase DNA damage, which could be alleviated by zinc repletion (48). Thus, zinc may preserve telomere length *via* reducing oxidative stress and inflammatory responses, increasing telomerase activity, and maintaining genomic stability.

Our study demonstrated that only females showed a significant correlation between zinc consumption and telomere length. However, this association was not found in males. This sex-related difference may be explained by women having better antioxidant status. A previous study reported that the level of estrogen was positively correlated with the expression level of antioxidant enzymes, such as superoxide dismutase and glutathione peroxidase, which suggested that estrogen could contribute to higher antioxidant activity in females (49). Moreover, a cross-sectional survey suggested that estrogen was responsible for zinc metabolism, which might explain the sex-related difference in telomere length as well (50). However, further studies are needed to confirm our hypothesis.

Additionally, we observed that elevated dietary zinc intake was significantly correlated with longer telomeres among obese rather than non-obese participants. Previous studies have indicated that participants with obesity had shorter telomere lengths than non-obese participants (51). Compared with non-obese people, people with obesity had higher inflammation and oxidative stress level (52). Besides, unhealthy lifestyles promoting obesity may exacerbate the status of oxidative stress in people with obesity (53). Moreover, it has been reported that zinc depletion was a risk factor for obesity, while zinc supplementation may play a potential therapeutic role for obesity (54). Therefore, we proposed that zinc played a significant protective role in the obese population *via* attenuating inflammation and oxidative stress.

Our study had several strengths. Firstly, as far as we know, this was the first epidemiological study to investigate the effect of dietary zinc intake on telomere length. Stratified analyses were further carried out to examine the possible modification effect of sex, BMI and overall energy intake. Our findings had significant clinical and public health implications for preventing chronic diseases and prolonging life. Secondly, our data were derived from NHANES 1999–2002 and the sample was large, multiracial, and randomly selected. Hence, our findings are broadly representative of the US adult population.

Nevertheless, our study also had some limitations that should be addressed in future studies. Firstly, the cross-sectional design of the NHANE survey failed to deduce the causal or temporal relationship between dietary zinc intake and telomere length. Secondly, recall bias and measurement errors were inevitable in 24-h dietary recalls. Thirdly, we cannot exclude the effects of other trace elements and vitamins (e.g. iron, copper, magnesium, selenium, riboflavin, and so on) and unmeasured factors (e.g. genetic factors) on the results. Finally, we did not explore the association between serum zinc levels and telomere length. Despite these limitations, our study could provide important clues about the roles of zinc in preventing telomere attrition.

## Conclusion

To conclude, our study elucidated a significant positive correlation between dietary zinc intake and telomere length in middle-aged and older adults in the United States. Our findings implied that adequate dietary zinc intake could protect against telomere attrition, particularly in females, obese, and low energy intake adults. And the observed effect of nutritional zinc intake was independent of overall energy intake. Additional large-scale prospective studies are required to confirm our findings.

## Data availability statement

Publicly available datasets were analyzed in this study. This data can be found here: https://www.cdc.gov/nchs/nhanes/ (NHANES 1999–2000 and 2001–2002).

## Ethics statement

The studies involving human participants were reviewed and approved by the Ethics Review Board of the NCHS (Protocol #98–12). The patients/participants provided their written informed consent to participate in this study.

## Author contributions

HS and YZ contributed to the conception and design of the study. HS performed the statistical analysis. XL, HY, HS, WS, and YL wrote the manuscript draft. XL, HY, HS, and YZ provided content and feedback to the manuscript and reviewed and edited the manuscript. All authors read and agreed to the published version of the manuscript.

## Footnotes

1
https://wwwn.cdc.gov/nchs/nhanes/Default.aspx
