# Supplementary material for: Potential effect of dietary zinc intake on telomere length: A cross-sectional study of US adults
Source: Front Nutr. 2022 Nov 16;9:993425. doi: 10.3389/fnut.2022.993425 (PMC9709254; doi:10.3389/fnut.2022.993425)
Supplement: Supplementary file 1 [file Table_1.DOCX]

**Supplementary table 1.** The correlation coefficients between zinc and other variables.

|  | Dietary zinc intake |
| --- | --- |
| Dietary iron intake | 0.74 |
| Dietary copper intake | 0.69 |
| Dietary magnesium intake | 0.72 |
| Dietary selenium intake | 0.70 |
| Dietary riboflavin intake | 0.72 |
